# An interpretable machine learning tool for predicting perioperative cardiac events in patients scheduled for hip fracture surgery: insights from the multicenter LUSHIP study

**DOI:** 10.1186/s44158-025-00291-6

**Published:** 2025-10-27

**Authors:** Danila Azzolina, Gianmaria Cammarota, Enrico Boero, Paola Berchialla, Savino Spadaro, Federico Longhini, Cristian Deana, Daniele Guerino Biasucci, Stefano D’Incà, Irene Batticci, Nicola Fasano, Edoardo De Robertis, Rachele Simonte, Salvatore Maurizio Maggiore, Valentina Bellini, Elena Giovanna Bignami, Luigi Vetrugno, LUSHIP Study Group, Irene Batticci, Nicola Fasano, Vito Marco Ranieri, Anna Pesamosca, Agnese Cattarossi, Saskia Granzotti, Alessandro Cavarape, Andrea Cortegiani, Lisa Mattuzzi, Luca Flaibani, Nicola Federici, Francesco Meroi, Marco Tescione, Andrea Bruni, Eugenio Garofalo, Mattia Bernardinetti, Felice Urso, Camilla Colombotto, Francesco Forfori, Sandro Pregnolato, Francesco Corradi, Federico Dazzi, Sara Tempini, Alessandro Isirdi, Moro Federico, Nicole Giovane, Milo Vason, Carlo Alberto Volta, Fabio Gori, Michela Neri, Auro Caraffa, Giovanni Cosco, Eugenio Vadalà, Demetrio Labate, Nicola Polimeni, Marilena Napolitano, Sebastiano Macheda, Angela Corea, Lucia Lentin, Michele Divella, Daniele Orso, Clara Zaghis, Silvia Del Rio, Serena Tomasino, Alessandro Brussa, Natascia D’Andrea, Simone Bressan, Giuseppe Neri, Pietro Giammanco, Alberto Nicolò Galvano, Mariachiara Ippolito, Fabrizio Ricci, Francesca Stefani, Lolita Fasoli, Piergiorgio Bresil, Federica Curto, Lorenzo Pirazzoli, Carlo Frangioni, Mattia Puppo, Sabrina Mussetta, Michele Autelli, Giuseppe Giglio, Filippo Riccone, Erika Taddei

**Affiliations:** 1 Biostatistics and Clinical Trial Methodology Unit, Clinical Research Center DEMeTra, Department of Translational Medicine, University of Naples Federico II, Naples, Italy; 2 Department of Translational Medicine, Università del Piemonte Orientale, Novara, Italy; 3 Anesthesia and General Intensive Care, Azienda Ospedaliero Universitaria Di Alessandria, Alessandria, Italy; 4 Anesthesia and Intensive Care Unit, San Giovanni Bosco Hospital, Turin, Italy; 5 Center of Biostatistics, Epidemiology and Public Health, Department of Clinical and Biological Sciences, University of Torino, Turin, Italy; 6 Department of Translational Medicine, Anesthesia and Intensive Care Unit, University of Ferrara, Ferrara, Italy; 7 Anesthesia and Intensive Care Unit, Department of Medical and Surgical Sciences, ‘Magna Graecia’ University of Catanzaro, Catanzaro, Italy; 8 Department of Anesthesia and Intensive Care, Health Integrated Agency of Friuli Centrale, Udine, Italy; 9 Department of Clinical Science and Translational Medicine, Tor Vergata’ University of Rome, Rome, Italy; 10 Anesthesia and Intensive Care Unit, Health Integrated Agency of Friuli Centrale, Tolmezzo Hospital, Tolmezzo, Italy; 11 Anesthesia and Intensive Care, Department of Medicine and Surgery, Università Degli Studi Di Perugia, Perugia, Italy; 12 Department of Innovative Technologies in Medicine and Dentistry, Gabriele d’Annunzio University of Chieti-Pescara, Chieti, Italy; 13 Critical Care Medicine and Emergency Department of Anesthesiology, SS. Annunziata Hospital, Chieti, Italy; 14 Department of Medicine and Surgery, University of Parma, Anesthesiology, Critical Care and Pain Medicine Division, Parma, Italy; 15 Department of Medical, Oral and Biotechnological Sciences, University of Chieti-Pescara, Chieti, Italy

**Keywords:** Machine learning, Perioperative risk, Major adverse cardiac events (MACE), Hip fracture surgery, Elderly patients, Lung ultrasound (LUS), Revised Cardiac Risk Index (RCRI), Predictive modeling; Clinical decision support

## Abstract

**Background:**

Elderly patients undergoing surgery for hip fractures are at high risk for perioperative Major Adverse Cardiac Events (MACE), which can markedly compromise postoperative outcomes. This study aims to develop a machine learning (ML) based, interpretable tool to predict MACE using clinical and ultrasound-based variables in this population.

**Methods:**

We analyzed data from 877 patients in the multicenter LUSHIP study, incorporating demographics, Revised Cardiac Risk Index (RCRI), functional status, and preoperative lung ultrasound (LUS) scores. Multiple ML models were trained and validated using bootstrap resampling. The final ensemble meta-model combined GBM (Gradient Boosting Machine) and GLMNET (Elastic-Net Regularized Generalized Linear Models).

**Results:**

The ensemble model achieved an AUROC of 0.86, with sensitivity and specificity of 0.72 and 0.83, respectively. These results significantly improve over traditional tools such as the Revised Cardiac Risk Index (RCRI), particularly when used alone. A significant contribution of this work is the integration of lung ultrasound (LUS) as a non-invasive, bedside biomarker, which notably improved risk prediction compared to the performance of the individual LUS marker alone (AUC = 0.78). Relevant predictors for the ML model are LUS score, RCRI score, and patient age. A web-based Shiny application was developed to enable real-time personalized risk estimation.

**Conclusion:**

This interpretable ML model improves perioperative cardiac risk stratification and profiling in elderly hip fracture patients and may guide targeted preventive strategies and resource allocation.

**Trial registration:**

CT04074876

**Supplementary Information:**

The online version contains supplementary material available at 10.1186/s44158-025-00291-6.

## Background

Hip fractures are among the most frequent and severe surgical emergencies in the elderly, representing a significant global public health concern. Patients aged 65 and older undergoing urgent hip fracture repair face a disproportionately high risk of perioperative complications, preeminent adverse cardiac events (MACE) such as myocardial infarction, heart failure, and arrhythmia [1]. These events are strongly associated with increased morbidity, prolonged hospital stay, and elevated mortality, making early risk identification relevant for improving outcomes and guiding preoperative planning [2].

Current cardiac risk stratification strategies, such as the Revised Cardiac Risk Index (RCRI), offer population-level estimates but often lack the granularity needed for individualized risk assessment, particularly in frail patients with complex comorbidities [3]. In recent years, point-of-care lung ultrasound (LUS) has emerged as a promising bedside tool to identify pulmonary pathology that may predispose patients to perioperative cardiac complications [4].

Preliminary studies suggest combining LUS with conventional risk indices may improve predictive performance [5]. However, practical tools for integrating all these data to tailor real-time patient care are lacking.

Artificial intelligence (AI) refers to the development of computer systems capable of performing tasks that typically require human intelligence, such as reasoning, decision-making, and learning, thereby simulating human behavior [6]. Nowadays, machine learning-based AI techniques are increasingly integrated into real life. In clinical research, interest in utilizing machine learning (ML) models for profiling patient outcomes has significantly increased [7–11]. The advantages of ML models rely on the possibility of processing massive amounts of complex clinical data and catching non-linear relationships and interactions often overlooked by classical statistical methods [12], leading to identifying patterns that may not be detectable through traditional approaches [13]. Indeed, advanced ML algorithms have been developed to predict the progression of diabetic kidney disease using big data, demonstrating the potential of ML in enhancing clinical decision-making [14]. Hence, integrating ML with current preoperative evaluation practice might represent a promising strategy for improving perioperative care [15]. From a clinical standpoint, an ML-based application that delivers actionable insights before surgery could support the implementation of individualized interventions, improving both preoperative optimization and the personalization of perioperative care [16]. Moreover, unlike traditional models that depend on static, population-level data, ML-driven tools can dynamically adjust to each patient’s physiological and clinical profile, thereby improving the precision and responsiveness of medical care [17]. This skill is clinically relevant in the perioperative setting, where patient conditions can rapidly change.​

Despite increasing interest in applying ML to perioperative care, its routine clinical adoption remains limited, primarily due to concerns regarding model transparency and interpretability [18]. Advancing clinical integration requires predictive tools that combine high performance with interpretability, ease of use, and practical applicability in real-time bedside decision-making.

In line with these requirements, we developed an interpretable ML-based tool for predicting MACE in elderly patients undergoing hip fracture surgery. Using data from the multicenter LUSHIP (Lung Ultrasound Score for the prediction of major adverse cardiac events in elderly patients undergoing HIP Surgery) study, we trained and compared multiple machine learning algorithms incorporating patient demographics, comorbidities, functional status, RCRI, and LUS scores. Our final ensemble model combines high predictive performance with interpretability through Local Interpretable Model-Agnostic Explanations (LIME) [19] and the Variable Importance via Personalized Odds Ratio (VIPOR) framework [20]. We implemented the model in a Shiny web application for real-time risk estimation to improve clinical usability.

## Method

### Study setting

A dataset of 877 patients from the LUSHIP database was analyzed for training and developing algorithms. The study is multicenter observational research aimed at addressing the challenges of preoperative risk stratification in elderly patients undergoing urgent orthopedic surgery. The research was conducted across 11 Italian universities and non-university hospitals; this prospective study explored the utility of preoperative lung ultrasound (LUS) scores as a predictive tool for major adverse cardiac events (MACE). Patient enrollment occurred between September 2019 and September 2020. All participating centers followed a standardized scanning protocol, and the clinicians performing LUS were trained in accordance with current guidelines [21, 22].

The inclusion criteria targeted patients aged 65 years or older who presented with hip fractures requiring surgery within 24 h of admission. Other details concerning the study can be found elsewhere [3].

### Statistical analysis

Data cleaning and preparation were conducted. These steps included importing the dataset, renaming the variables for consistency, and recording the variables for analytical purposes. Specifically, categorical variables, such as ECG results, smoking status, and comorbid conditions, were recorded as binary or categorical, as appropriate.

MACE was defined as a new onset of atrial fibrillation requiring cardiologist consultation, as well as the occurrence of heart failure, acute myocardial infarction, or cardiac arrest [23].

Continuous variables were summarized using medians and interquartile ranges, whereas categorical variables were summarized using frequencies and percentages. The association between each predictor and MACE outcome was assessed and reported via univariable logistic regression Odds Ratio (OR), with 95% confidence intervals. A descriptive analysis comparing major adverse cardiac events (MACE) across different patient characteristics and clinical measures was reported elsewhere in the LUSHIP main publication [3].

### Machine learning models

Machine learning models were trained to predict the occurrence of MACE, including Random Forest (RF), Gradient Boosting Machines (GBM), Generalized Linear Model (GLM), GLM via penalized maximum likelihood (GLMNET), Support Vector Machines with linear (SVM) and radial basis function kernels (SVMR), Multilayer Perceptron (MLP), simple Neural Networks (NNET), and a Deep Learning (DL) neural network structure. Models were assessed with performance metrics such as the Area Under the Receiver Operating Characteristic (AUROC). The model AUROC has been compared with the classical RCRI index for preoperative risk by using a DeLong test [24]. The 95% CI for AUROC, sensitivity, and specificity of the final model was obtained via 500 bootstrap resamples. The final AUROC values reported are optimism-corrected estimates obtained from the resampling procedure. Threshold selection was based on Youden’s J and determined within the bootstrap framework to avoid data leakage. Furthermore, resampling was stratified by outcome to preserve the event/non-event ratio across samples. Model calibration was also assessed using the calibration slope, the Brier score, and the expected calibration error (ECE) based on decile binning.

A description of the machine-learning model is included and reported in the supplementary material.

### Training

Model training was guided using a bootstrapping method with 100 resampling iterations to estimate the accuracy of the models. The data was pre-processed by centering and scaling to improve the model's performance. An oversampling procedure was conducted to handle the class imbalance during the training phase. The predictive performance of the models was compared using resampling statistics, and visualizations were generated to compare the distribution of AUROC values across the models, highlighting the variability and predictive capacity of each approach.

### Validation

The internal validation process involved evaluating the ability of the models to accurately distinguish between patients with and without significant adverse cardiac events. This was accomplished by analyzing the models' AUROC, sensitivity (true positive rate), and F1 scores (harmonic mean of precision and sensitivity).

Violin plots were used to visualize the distribution of the AUROC and sensitivity values across the different models, providing insights into the model’s performance variability. Additionally, the calculation of F1 scores offered a balanced measure of the model's precision and recall, with the results depicted in a corresponding violin plot to facilitate comparison.

### Model ensembling

The final phase of the analysis involved creating an ensemble meta-model that combined the predictions of the leading-performing models. This ensemble approach aims to improve the strengths of individual models, potentially offering better performance by reducing the risk of overfitting the training data and capturing a broader representation of the data patterns.

Initially, separate predictive models are trained and internally validated. After that, the best-performing single models are combined into a list of models that integrate them into a single ensemble. This is achieved by using the predictions from the individual models as input features for a new metamodel. The metamodel is then trained to predict the outcome based on these inputs. In the stacking approach, each base model in the ensemble makes predictions for the same dataset. These predictions are then used as input features for the GBM metamodel. Essentially, the GBM metamodel learns how to best combine the predictions to obtain a final one. During training, the GBM focused on optimizing the AUROC in 100 iterations. Thus, it learns the optimal weighting for predictions from the base models.

The efficacy of the ensemble model was evaluated using AUROC curve analysis to identify the optimal threshold for predicting major adverse cardiac events. Examination of the ensemble model's predictions provided results regarding the sensitivity, specificity, accuracy, and optimal cut-off point of MACE probability.

### ML tool interpretation

Variable importance plots were generated to identify the most influential predictors in the model, thus enhancing the interpretability of the ensemble model's decision-making process. The partial dependency plot for the leading predictors is reported in the Supplemental Digital Content.

This study employed the local interpretable model-agnostic explanation (LIME) methodology to shed light on the decision-making processes of complex machine learning models predicting MACE. This analysis focused on elucidating the model's predictions for selected patient records, providing a granular view of the influential factors driving these predictions. This approach highlighted the key variables impacting the model's predictions. It quantified their contribution, offering some interpretative findings into their relative importance and direction of influence (positive or negative) regarding the outcome of interest. The explanations were visualized using a plot function, producing intuitive plots that graphically represented the contribution of each feature to the prediction.

The variable importance evaluation with the personalized odds ratio [20] tool was also considered for reporting the covariate effect on the final predictor. In our statistical analysis section, we use Variable Importance based on the Personalized Odds Ratio (VIPOR) framework to assess the importance of variables within nonlinear machine learning models. This model-agnostic method bridges the gap between the predictive properties of complex algorithms and the necessity for interpretable insights, which are crucial in the clinical and biomedical domains.

The VIPOR method, grounded in the well-established odds ratio (OR) concept, facilitates both local and global interpretations of the model predictions. Locally, it quantifies variable importance through personalized odds ratio (POR), accommodating individual subject heterogeneity. Globally, a hierarchical structure groups variables into five distinct categories based on their POR (Personalized Odds Ratio) distributions, ranging from consistently positive or negative effects to more nuanced, mixed, or dominant effects. This categorization enabled us to define the variable's impact across the population, providing insights into their general behavior and influence on the predictive model.

The variables were categorized as follows: 1) positive if the POR was > 1 for all the subjects included in the model, 2) positive dominated if the POR was > 1 for at least 50% of patients, 3) negative if the personalized POR was < 1 for all subjects included in the model, 4) negative dominated if the POR was < 1 for at least 50% of patients, and 5) negligible importance variable in the other cases. The mean POR per variable is reported using a VIPOR plot. Details concerning the POR calculation are reported in the Supplemental Digital Content.

### Shiny web app

A shiny web application (https://biostatlab.shinyapps.io/LushipPredictioneR/) was developed to obtain a final ensemble model that predicted MACE score probability according to a user-defined patient profile. Users can typically manipulate inputs on a patient's characteristics to see how they influence the MACE probability output in real time. Further details concerning the web app are provided in the Supplementary Material section.

Computations have been performed using the Caret [25] package in R [26] (version 4.0.1), and the Tensorflow [27] package in R interfacing with Python [28] (version 9.3). The web app was developed in the Shiny environment.

## Results

### Univariable associations with MACE risk

The dataset comprised 877 patients, with 779 (89%) having no MACE and 98 (11%) experiencing such events [3].

Table 1 reports the univariable MACE OR (Odds Ratio) of the patients' characteristics considered to train the ML models. Other descriptive details are reported elsewhere [3].

**Table 1:** Univariable logistic regression OR (odds ratio) table of the variables considered for the ML training algorithms

	**OR**	***P*-value**
**Gender**		
Female	Ref	Ref
Male	1.05 [0.64;1.66]	0.854
**Age (year)**	1.01 [0.98;1.03]	0.634
**BMI**	1.02 [0.98;1.07]	0.264
**Time to surgery (days)**	1.03 [0.96;1.11]	0.368
**Smoke**		
No	Ref	Ref
Yes	1.67 [0.93;2.85]	0.084
**Acute coronary heart disease**		
No	Ref	Ref
Yes	2.85 [1.76;4.53]	< 0.001
**ASA score**	2.11 [1.48;3.00]	< 0.001
**CHF**		
No	Ref	Ref
Yes	5.60 [3.38;9.17]	< 0.001
**Creatinine**		
> 1.5/mgdl	Ref	Ref
< = 1.5/mgdl	0.27 [0.17;0.43]	< 0.001
**Diabetes**		
No	Ref	Ref
Yes	2.15 [1.24;3.61]	0.008
**Stroke**		
No	Ref	Ref
Yes	1.60 [0.87;2.81]	0.128
**Functional status**		
Independent	Ref	Ref
Partially independent	1.27 [0.79;2.03]	0.323
Dependent	2.01 [1.11;3.57]	0.023
**Hypertension**		
No	Ref	Ref
Yes	1.02 [0.57;1.71]	0.956
**LUS score**	1.20 [1.16;1.25]	< 0.001
**SID**		
No	Ref	Ref
Yes	5.24 [3.06;9.63]	< 0.001
**RCRI Score**		
0–1	Ref	
2–4	5.73 [3.50;9.30]	

A significant increase in risk of MACE is evidenced for patients with scores of 2–4 RCRI compared with scores of 0–1. Smoking status and the presence of comorbidities such as coronary artery disease (CAD), congestive heart failure (CHF), and diabetes were significantly associated with MACE. Specifically, patients with these conditions are more likely to experience adverse events. The total lung ultrasound score (LUS total) and SID status (Severe Ischemic Disease) were strongly associated with MACE, indicating that patients with higher LUS scores and those classified as SID had significantly higher odds of experiencing MACE. Functional status was also a significant predictor, with those entirely dependent having higher odds than those who were independent.

### Machine learning – stacked predictive modeling training and validation

Validation processes for the AUROC, sensitivity, and F1 scores were conducted to ensure the robustness and reliability of the single predictive models. These processes involved resampling techniques and comparing model performance metrics to identify the most effective predictors of MACE.

Figure S1 illustrates the distribution of the AUROC values across different predictive models within training resampling. Each violin shape represents the distribution of the AUROC values for one model, highlighting the data's density at different values and the distribution range. The GBM and GLMNET models showed similar distributions and central tendencies for AUROC values, peaking around 0.778. The RF model's distribution was slightly wider, indicating more variability in the AUROC values, with a median of approximately 0.770. Overall, the GBM and GLMNET models are the top performers based on the median AUROC values, indicating a higher discriminative ability for any outcome they predicted. Figure S2 (Supplementary Material) indicates that GLMNET had the highest median sensitivity value of 0.667, suggesting that it is the best among the compared models for identifying true positives. The distributions for GBM and GLM were similar, with median sensitivity values of 0.618 and 0.600, respectively. Based on the violin plot, GLMNET and GBM were the most sensitive models.

The distribution of the F1 scores for the different predictive models is shown in Fig. S3 (Supplementary Material). The F1 score is a harmonic mean of precision and recall that balances a model's ability to correctly label positives and the completeness of positive predictions. GLMNET was at the top, with a median F1 score of 0.721, indicating a strong balance between precision and recall. The GBM machine follows closely, with a median F1 score of 0.695, suggesting that it also effectively balances the positive predictive value and sensitivity. The plot suggests that while all models have some predictive value, some (such as GLMNET and GBM) are more consistently reliable in their predictive performance for the task at hand. For this reason, the GLMNET and GBM models were selected to develop the ensemble prediction model.

### Ensemble meta model assessment

An ensemble model combining predictions from the GBM and GLMNET models showed a significant predictive capability. The relative importance within the ensemble model was 69.94% for GLMNET and 30.06% for GBM, indicating the predominant influence of the GLMNET model in the ensemble predictions (Fig. S4, Supplementary material).

### Final meta-model performance

**Fig. 1:**
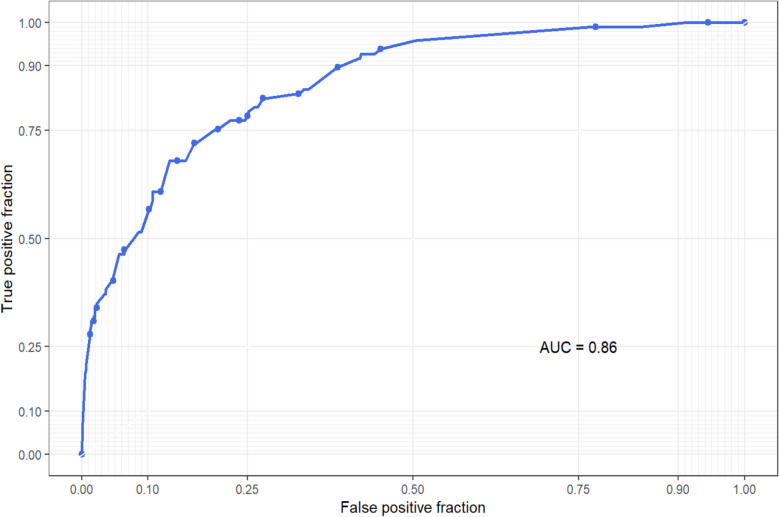
ROC (Receiving Operating Characteristic) curve for the ML tool final meta model ensemble. The sensitivity is 0.72 (95% CI = 0.62–0.87) and a specificity of 0.83 (95% CI = 0.66–0.91) at an optimal cut-off point of 0.604 achieved by Youden Threshold

Figure 1 shows the AUROC curve for the ensemble final meta model. The curve's progression from the bottom left to the top left and then toward the top right corner suggests that the ensemble model has a good balance between sensitivity and specificity with an AUROC of 0.86 (95% CI = 0.84–0.88), while the RCRI alone showed an AUROC of 0.69 (95% CI = 0.63–0.74). The formal comparison yielded a statistically significant difference (*p* < 0.001). The sensitivity is 0.72 (95% CI = 0.62–0.87) and a specificity of 0.83 (95% CI = 0.66–0.91) at an optimal cut-off point of 0.604 achieved by Youden Threshold.

The model achieved a Brier score of 0.15, indicating good overall accuracy. The calibration slope was 1.29, suggesting a slight tendency toward overconfident predictions. The expected calibration error (ECE) was 0.24, pointing to moderate miscalibration between predicted and observed event rates.

### Model interpretation

**Fig. 2:**
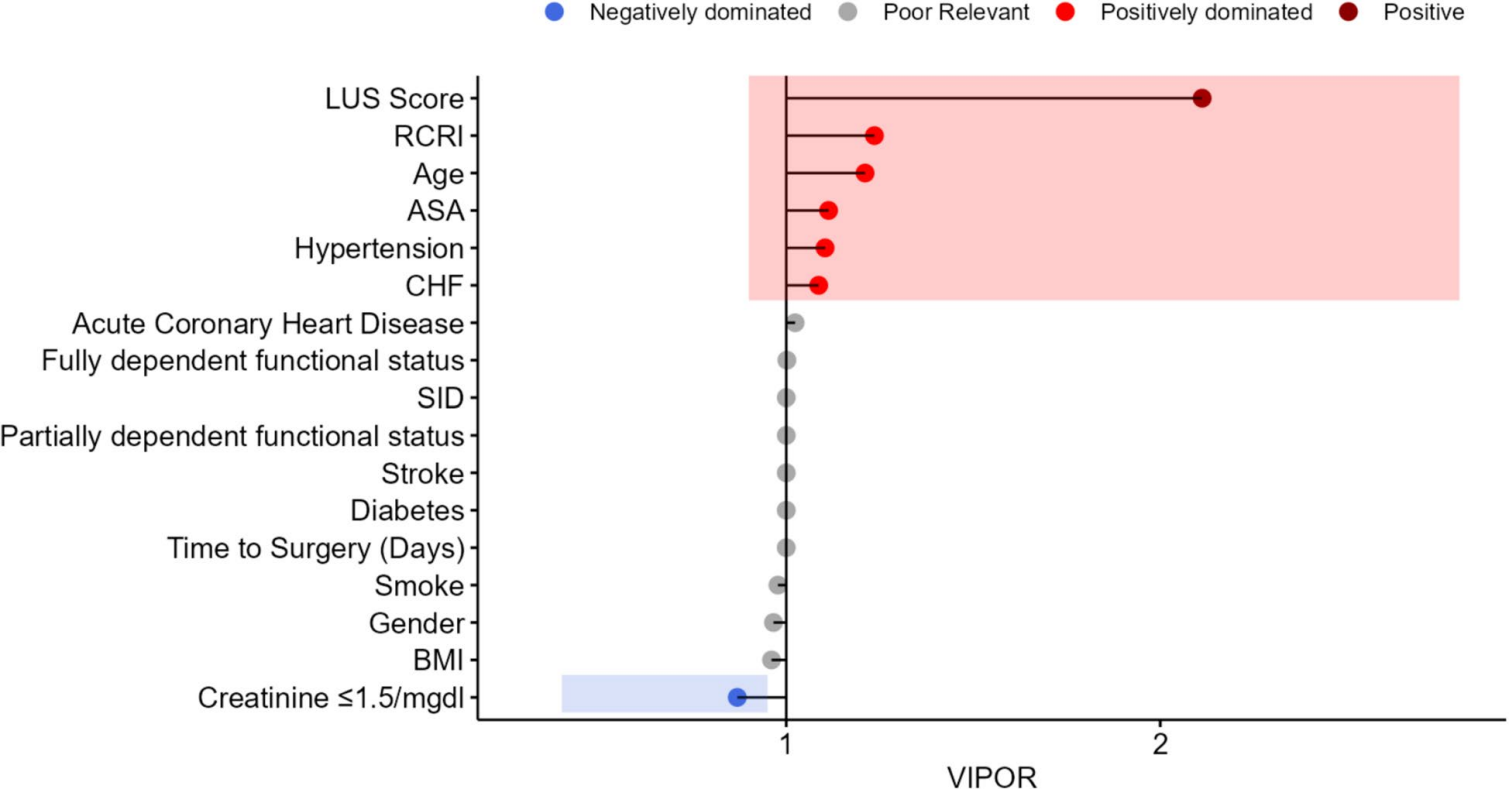
VIPOR plot. The x-axis shows the VIPOR values that measure the impact of each variable on the model's predictions on the OR scale. A VIPOR value greater than 1 indicates a variable that tends to increase the probability of the predicted outcome when it is present (positively dominated). In contrast, a value less than 1 indicates the opposite effect (negatively dominated). The y-axis shows the variables used in the model. These include clinical variables like LUS Score (Lung Ultrasound Score (LUS), Revised Cardiac Risk Index (RCRI), patient demographics such as age and sex, and medical history factors such as Acute Coronary Heart Disease and Stroke. The dots were color-coded to indicate the dominance type. Red indicates positively dominated variables, suggesting that these factors significantly increased the likelihood of the outcome being predicted. Dark grey indicates variables with poor relevance that do not have a strong impact on the model's predictions. Blue indicates negatively dominated variables, suggesting that these factors decreased the likelihood of the outcome. The plot also includes a shaded area (red rectangle), which seems to highlight the positively dominated variables. This indicates that these are variables of interest or concern in the context of the analysis

The VIPOR plot is reported in the web app in Fig. 2. The figure reveals that variables such as the LUS Score, RCRI, and age are among the most positively impacted on the model's predictions. This indicates that they were significant contributors to the predicted outcome. In contrast, Creatinine ≤ 1.5 mg/dl appears to be a negatively dominated variable, implying that it reduces the likelihood of the expected result when higher than its range. The classical variable importance plot (Fig. S5, Supplementary material) identified the same variables as relevant, indicating consistency between the two methods in identifying key predictors. Partial dependency plots of the leading predictors are shown in Fig. S6, indicating an increase in the predicted probability of MACE as the LUS score (Panel A) and RCRI (Panel B).

### Shiny web application

A Shiny web application has been developed (https://biostatlab.shinyapps.io/LushipPredictioneR/). The user could open the link and insert the patients’ characteristics as depicted in Fig. S6. Once the characteristics have been selected and the calculate button selected, a plot reporting the predicted probability of MACE for the patient profile is reported, together with the LIME importance plot for the specific patient profile, for example, in (Fig. S7, Supplement).

The first image displays a bar graph indicating the predicted probability of experiencing a major adverse cardiac event (MACE). There are two bars, each representing a different status: This bar is approximately 0.755, suggesting a 75.5% probability of the event occurring. The second bar indicates a 24.5% probability that MACE will not happen.

In the second plot, the features that increase the likelihood of the predicted outcome "MACE" are shown in blue as "Supports", while those that decrease it are in red as "Contradicts".

- The LUS Score being greater than 8 is the most significant contributor to increasing the prediction probability, indicating a strong influence on the model predicting the occurrence of MACE.
- A RCRI greater than 1 also supports the prediction, but with less weight than the LUS Score.
- Being of an Age less than or equal to 75 contradicts (reduces the likelihood of) the occurrence of MACE.
- Hypertension present (equals 1) has a supporting effect, albeit smaller in comparison to the LUS Score and RCRI.
- The ASA score ranging from 2 to 3 has the least influence among the listed features but still supports the prediction.

## Discussion

In this multicenter study, we developed and internally validated an interpretable machine learning–based model for predicting perioperative major adverse cardiac events (MACE) in elderly patients undergoing hip fracture surgery. The ensemble final model achieved strong predictive performance with an AUROC of 0.86. These results significantly improve over traditional tools such as the Revised Cardiac Risk Index (RCRI), particularly when used alone. A significant contribution of this work is the integration of lung ultrasound (LUS) as a non-invasive, bedside biomarker, which notably enhanced risk prediction compared to the performance of the individual LUS marker alone (AUC = 0.78) [3]. Moreover, single markers do not allow for personalized risk profiling based on patient characteristics when used in isolation, as is possible with an integrated machine learning approach [3, 29].

Assessing the importance of clinical predictors, LUS scores emerged in our model as one of the most influential variables, alongside age and RCRI, confirming previous results indicated in the literature [3] and supporting its integration into preoperative evaluation pathways, as recently recommended in international guidelines. Including the LUS score among the main predictive factors of the model is particularly relevant. Indeed, preoperative lung ultrasound assessment has proven helpful in identifying any subclinical pulmonary disease, such as interstitial syndrome, a condition strongly associated with an increased MACE risk [30]. This aligns with recent guidelines, emphasizing the utility of point-of-care lung ultrasound in preoperative evaluation for non-cardiac surgery with a final suggestion supporting its integration into preoperative workflow [31].

From the clinical standpoint, the RCRI and patient age emerged as relevant contributors to MACE prediction, reaffirming their central role in perioperative risk stratification. This finding aligns with longstanding evidence that age is a dominant, independent predictor of adverse perioperative outcomes, particularly in the elderly undergoing urgent orthopedic surgery [1]. With advancing age, the prevalence of cardiac comorbidities, frailty, and physiological reserve decline significantly, all of which contribute to heightened vulnerability to hemodynamic stress and myocardial ischemia during the perioperative period [32].

Similarly, the RCRI has been widely adopted as a benchmark risk assessment tool, particularly for non-cardiac surgery [33]. However, its use alone offers only a coarse estimate of risk at the population level and may not sufficiently capture patient-specific nuances, especially in frail or polymorbid elderly patients [34]. The model uses its known prognostic utility by incorporating RCRI into a multivariable machine learning framework while augmenting it with dynamic, patient-specific features. This integrative approach underscores the limitations of relying on single indices and highlights the need for multifactorial, individualized risk-stratification strategies [34].

Moreover, the interplay between LUS, RCRI, age, and other patient characteristics within the model suggests that these variables do not act in isolation but contribute additively or interactively within a broader clinical context. This reinforces a shift in perioperative medicine toward composite predictive frameworks that integrate demographic, functional, and physiological domains to inform tailored interventions [16].

Building on this foundation, machine learning offers complementary potential in perioperative medicine, improving outcome prediction, optimizing anesthesia planning, enabling real-time monitoring, and supporting more efficient, cost-effective clinical decision-making [16, 17]. Machine learning has demonstrated considerable versatility in forecasting a range of specific perioperative complications, including post-induction hypotension, postoperative major adverse cardiac events (MACE), acute kidney injury, and major bleeding [35–40]. A substantial advancement in ML is the surgical Variational Autoencoder (surgVAE) model, which utilizes advanced representation learning to predict multiple complications in cardiac surgery [41]. SurgVAE achieved macro-averaged AUCs of 0.831, surpassing traditional and advanced ML models in providing clear risk profiles [41]. Despite these improvements, in anesthesiology, intensive care, and pain medicine, the implementation of ML-based technology is still irregular and sporadic due to a lack of usability and interpretability [42].

Instead, the present study represents a new attempt to spread knowledge on ML applied to anesthesia and perioperative medicine. It provides anesthesiologists with an ML-driven tool to support their decision-making processes and improve patient outcomes, particularly in elderly patients scheduled for hip fracture surgery.

The development of a Shiny-based web application improves usability, offering physicians a real-time and interpretable decision support tool. Our ensemble model offers a more interpretable and clinically adaptable alternative for tabular data environments daily in perioperative settings compared to deep learning models, which may require large datasets and remain opaque in their decision-making [43].

To address the challenge of clinical interpretability, often cited as a barrier to adopting machine learning in healthcare, we applied the VIPOR [20] framework to estimate personalized odds ratios and the LIME [19] method to explain individual predictions visually. This interpretability allows clinicians to understand which variables drive the predicted risk, building trust and facilitating clinical adoption.

These results point out the ability of ML techniques to rely on integrating multidimensional data to provide accurate predictions. From the technical standpoint, the ensemble of GBM and the GLMNET outperform the more complex DL approaches; GBM and GLMNET are particularly small-medium-sized tabular datasets with a limited number of patient characteristics, whereas DL, while powerful in domains like image recognition and natural language processing, in several contexts can suffer of overfitting acting as a "black box" and requiring extensive data and tuning to generalize well [44]. Furthermore, these methods are computationally efficient, requiring standard CPUs instead of specialized hardware like GPUs, making them practical for resource-constrained environments [45]. This research, moreover, incorporates some interpretative tools, offering clinicians a window into the influences each variable has on MACE outcomes beyond the “black box” [20]. Coupled with a Shiny app, this research provides healthcare professionals an interactive platform to predict MACE, making our findings statistically significant and clinically actionable.

### Study limitations

This study is not without limitations. Despite promising results, the model was developed on a dataset from Italian hospitals, which may limit generalizability to other health systems. Therefore, external validation of diverse populations is needed in future research developments. Due to the progressive introduction of AI device technology in perioperative medicine, future research should focus on improving the predictive performance of ML algorithms by timely integrating the data stream conveyed by these tools. In addition, although Decision Curve Analysis (DCA) could further clarify the clinical net benefit of the model, we deliberately focused this work on model development, interpretability, and internal validation; we plan to address DCA in future external validation studies to better illustrate its impact on clinical decision-making.

Moreover, patients undergoing hip fracture surgery represent a heterogeneous population influenced by both measured and unmeasured factors, which may affect model development; this heterogeneity should be acknowledged [46], and future studies are needed to explore potential subgroup-specific differences in outcomes and model performance.

Future directions include external validation, EHR integration, and expansion to include intraoperative and postoperative data or wearable sensor inputs to improve dynamic risk modeling. Moreover, the model was built on MACE definitions adopted in the LUSHIP study [3]. Accordingly, due to the abovementioned bias, the performance and accuracy of the present model could be different in predicting the onset of MACE, which is defined differently.

## Conclusion

This study highlights the potential of machine learning to improve perioperative cardiac risk stratification in elderly patients undergoing hip fracture surgery. We developed an interpretable ML-based model that provides accurate, individualized risk estimates by combining clinical variables with lung ultrasound data. Implemented in a user-friendly web application, the tool offers real-time support for preoperative decision-making, enabling more personalized and informed perioperative management in this high-risk population.

## Supplementary Information

Supplementary Material 1.

## Data Availability

De-identified data may be available from the corresponding author upon reasonable request to the corresponding author and with appropriate institutional approvals.

## Abbreviations

AI: Artificial Intelligence
AKI: Acute Kidney Injury
ASA: American Society of Anesthesiologists
AUROC: Area Under the Receiver Operating Characteristic Curve
CHF: Congestive Heart Failure
DL: Deep Learning
ECG: Electrocardiogram
GBM: Gradient Boosting Machine
GLM: Generalized Linear Model
GLMNET: Elastic-Net Regularized Generalized Linear Model
ICU: Intensive Care Unit
LIME: Local Interpretable Model-Agnostic Explanations
LUS: Lung Ultrasound
MACE: Major Adverse Cardiac Events
ML: Machine Learning
MLP: Multilayer Perceptron
NNET: Neural Network
OR: Odds Ratio
POR: Personalized Odds Ratio
RCRI: Revised Cardiac Risk Index
RF: Random Forest
SID: Severe Ischemic Disease
SVM: Support Vector Machine
SVMR: Support Vector Machine with Radial Kernel
VIPOR: Variable Importance based on Personalized Odds Ratio
