# Supplementary material for: An interpretable machine learning tool for predicting perioperative cardiac events in patients scheduled for hip fracture surgery: insights from the multicenter LUSHIP study
Source: J Anesth Analg Crit Care. 2025 Oct 27;5:71. doi: 10.1186/s44158-025-00291-6 (PMC12560482; doi:10.1186/s44158-025-00291-6)
Supplement: Supplementary file 1 — Supplementary Material 1. [file 44158_2025_291_MOESM1_ESM.docx]

# Supplementary Material

## Algorithms

**Random Forest (RF)**: A Random Forest is an ensemble technique that constructs multiple decision trees during training and outputs the average prediction of the individual trees for regression tasks or the majority vote for classification. It is known for its high accuracy, robustness to overfitting, and ability to handle large datasets with higher dimensionality[1].

**Gradient Boosting Machines (GBM)**: GBM iteratively constructs new models that are added to correct the errors made by existing ensembles. It is a highly effective and widely used machine learning algorithm that has found success in a range of applications, notably in structured or tabular data[2].

**Generalized Linear Model (GLM)**: GLMs extend linear models by allowing for the response variable to have a non-normal distribution and for the linear model to be related to the response through a link function. GLMs are used in various fields for regression analysis[3].

**GLM via penalized maximum likelihood (GLMNET)**: GLMNET is an extension of GLMs, which includes regularization terms in the loss function (L1 and L2 penalties) to prevent overfitting and allow for variable selection, particularly useful when dealing with datasets with many features[4].

**Support Vector Machines (SVM)**: SVM is a powerful classification technique that finds a hyperplane in an N-dimensional space that distinctly classifies the data points. It is well-regarded for its effectiveness in high-dimensional spaces and its versatility, as it can be used with a variety of kernel functions[5].

**Support Vector Machines with Radial Basis Function kernels (SVMR)**: SVMR is a variation of SVM that uses a radial basis function kernel to map input features into high-dimensional space where linear separation is possible, particularly effective for non-linear relationships[6].

**Multilayer Perceptron (MLP)**: MLP is a type of neural network consisting of multiple layers of neurons, each layer fully connected to the next one. MLPs can approximate virtually any continuous function and are particularly known for their capability in feature learning[7].

**Simple Neural Networks (NNET)**: NNETs typically refer to neural networks with one or a few layers, which can capture complex relationships in the data but are less computationally demanding than deep learning models[8].

**Deep Learning (DL) Neural Network Structure**: Deep Learning models are composed of multiple processing layers to learn representations of data with multiple levels of abstraction. These models have been used to achieve state-of-the-art results in fields like image and speech recognition[9].


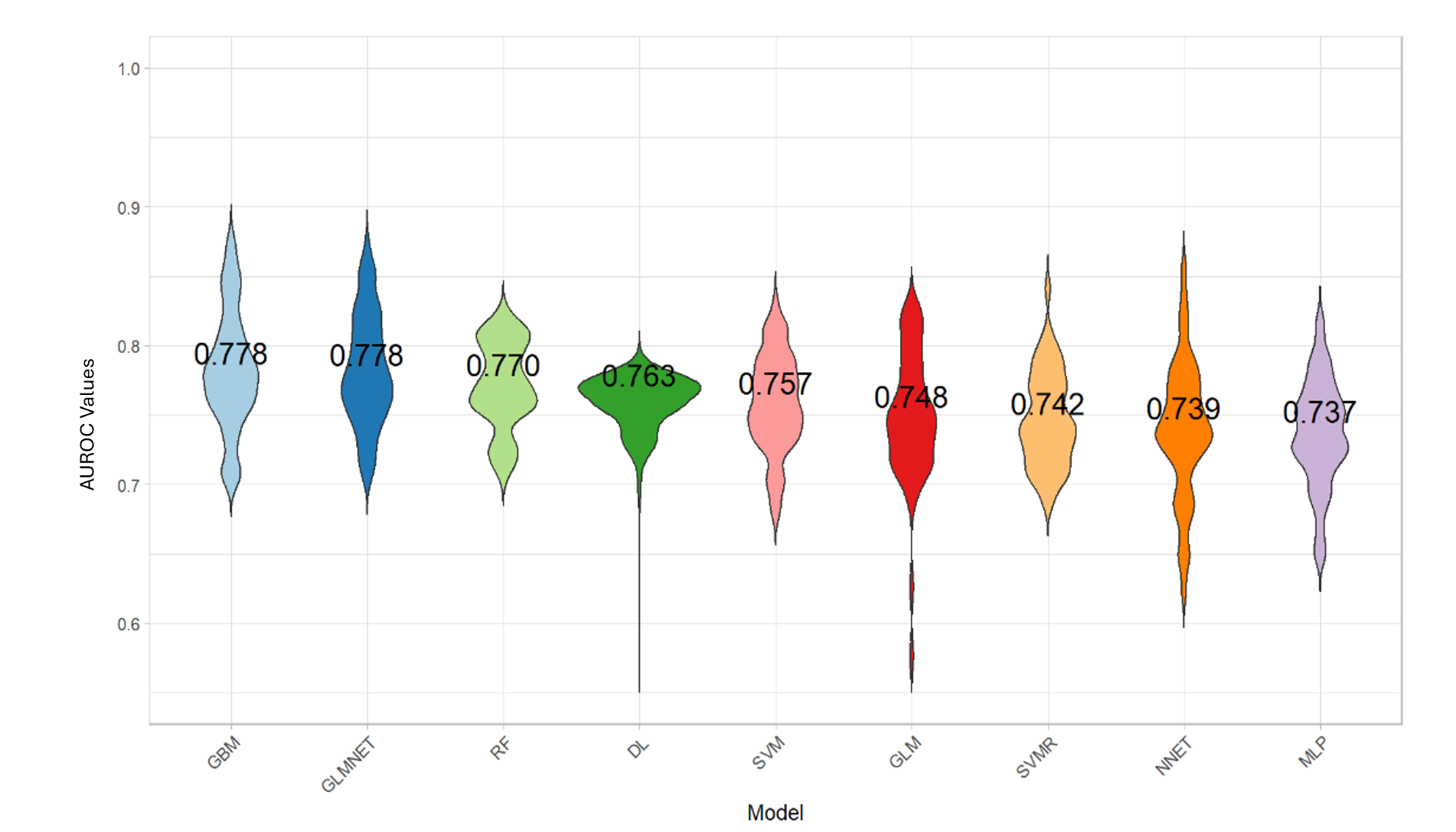


Figure S1 AUROC Value according to trained ML within bootstrap validation resampling. The median values are reported in the plots.


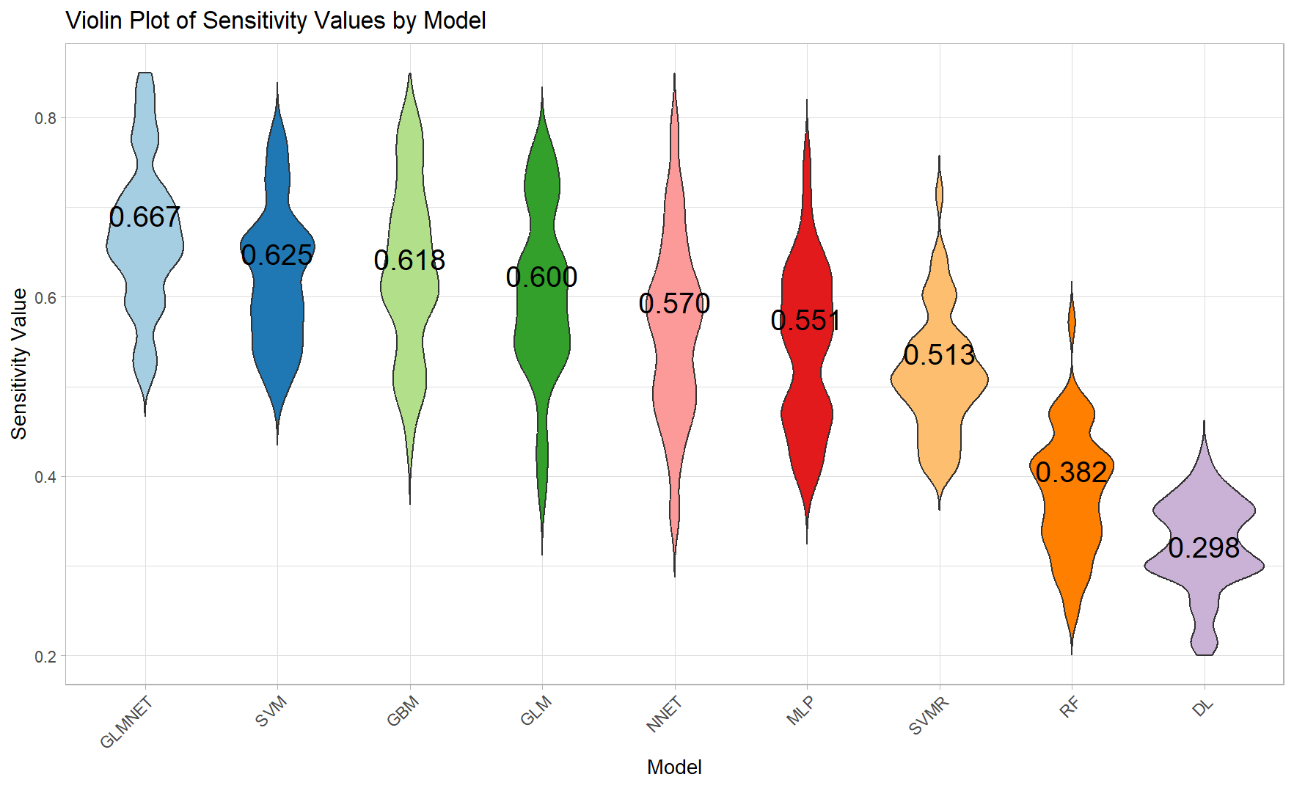


Figure S2 Sensitivity Value according to the trained ML within bootstrap validation resampling. The median has been reported on the plots.


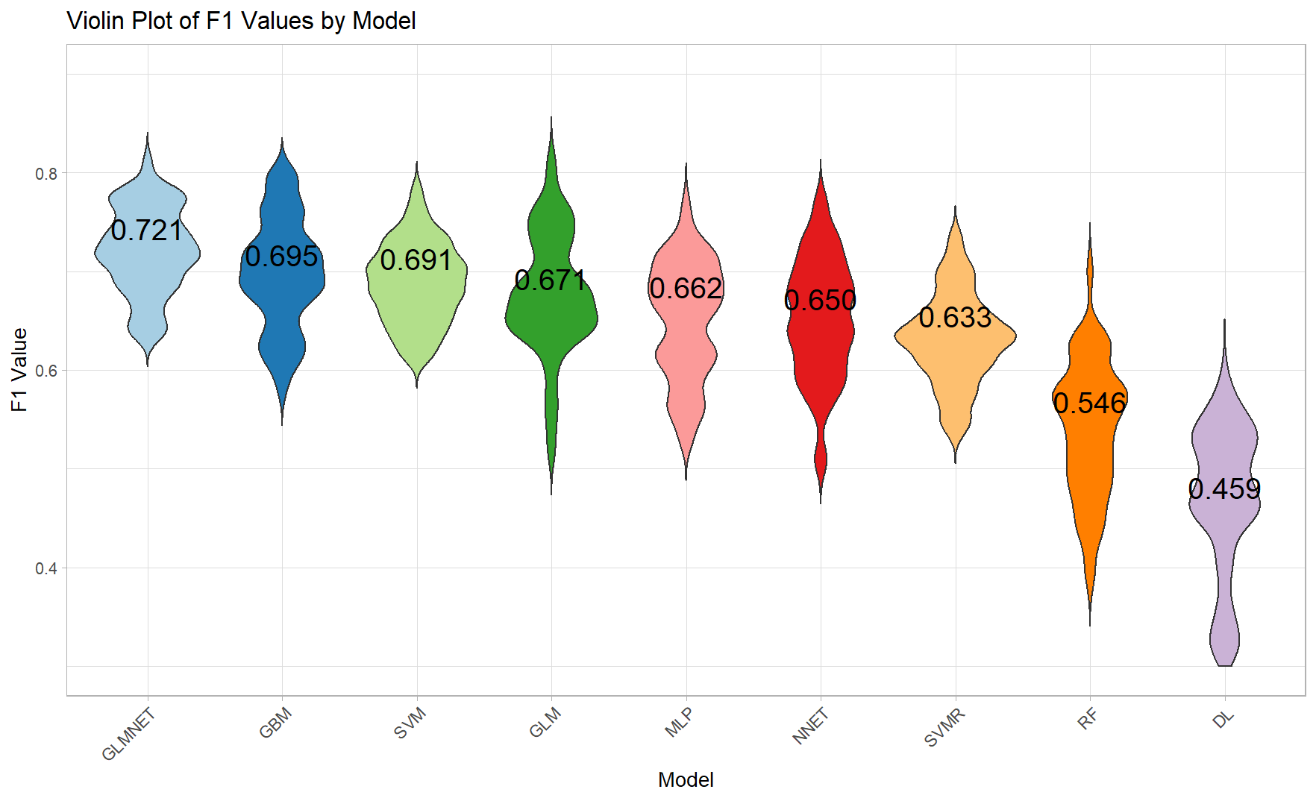


Figure S3 F1 Value according to the trained ML within bootstrap validation resampling. The median has been reported on the plots.


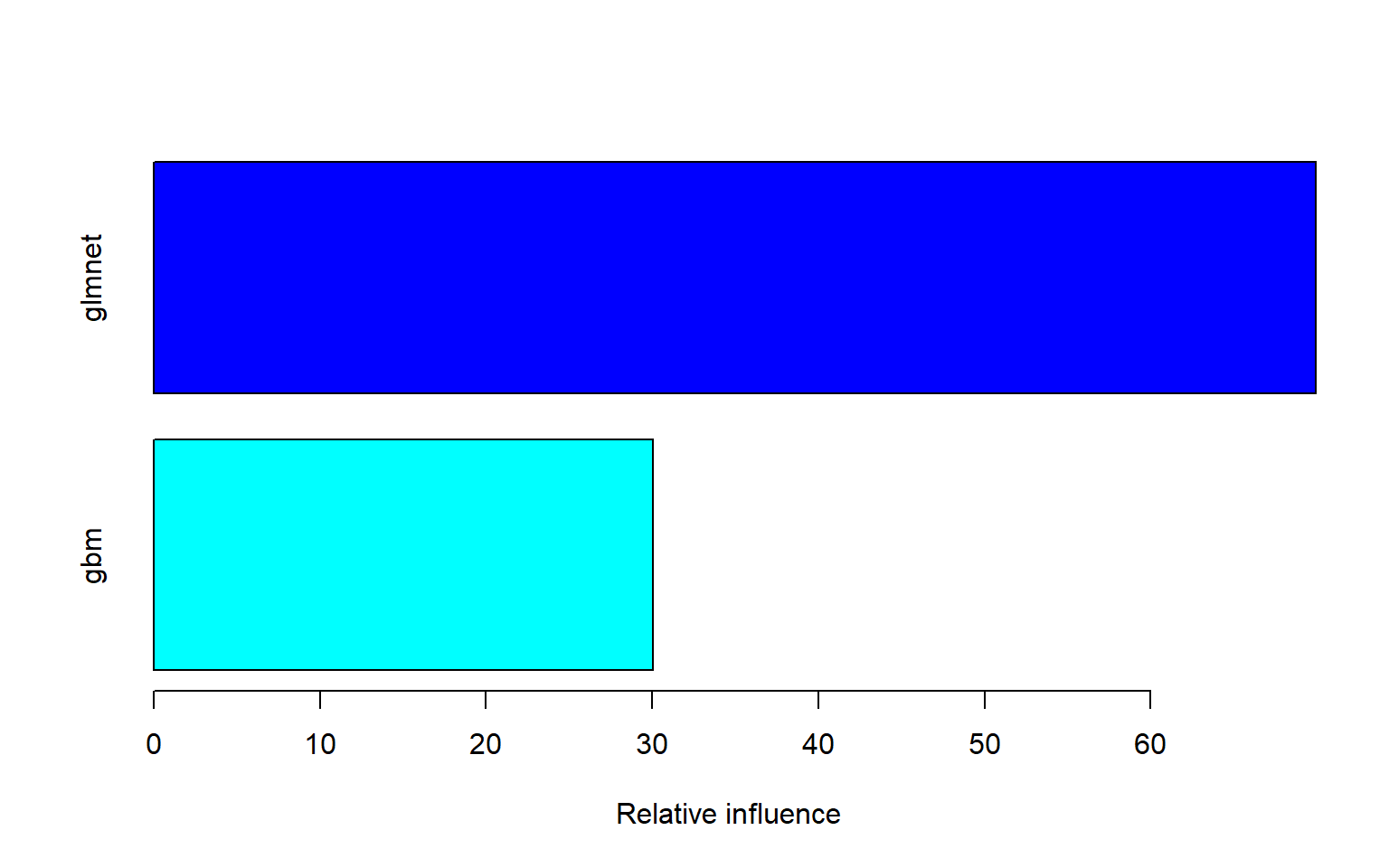


Figure S4 Relative influence plot for the algorithms composing the ensemble machine.


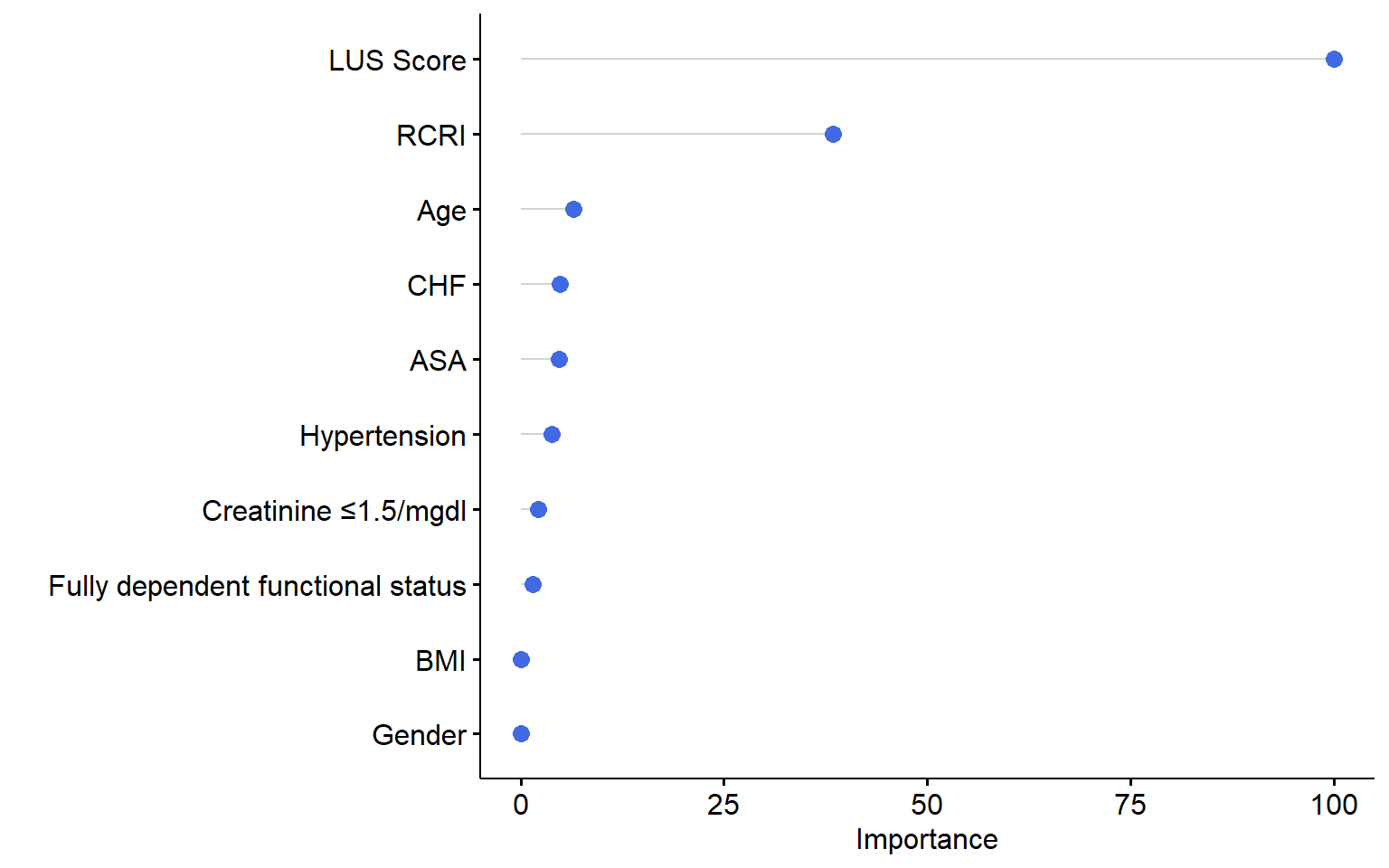


*Figure S5 Variable importance relative weight of the leading machine (GLMNET) composing the ensemble.*

| *Panel A Partial Dependency plot LUS*  *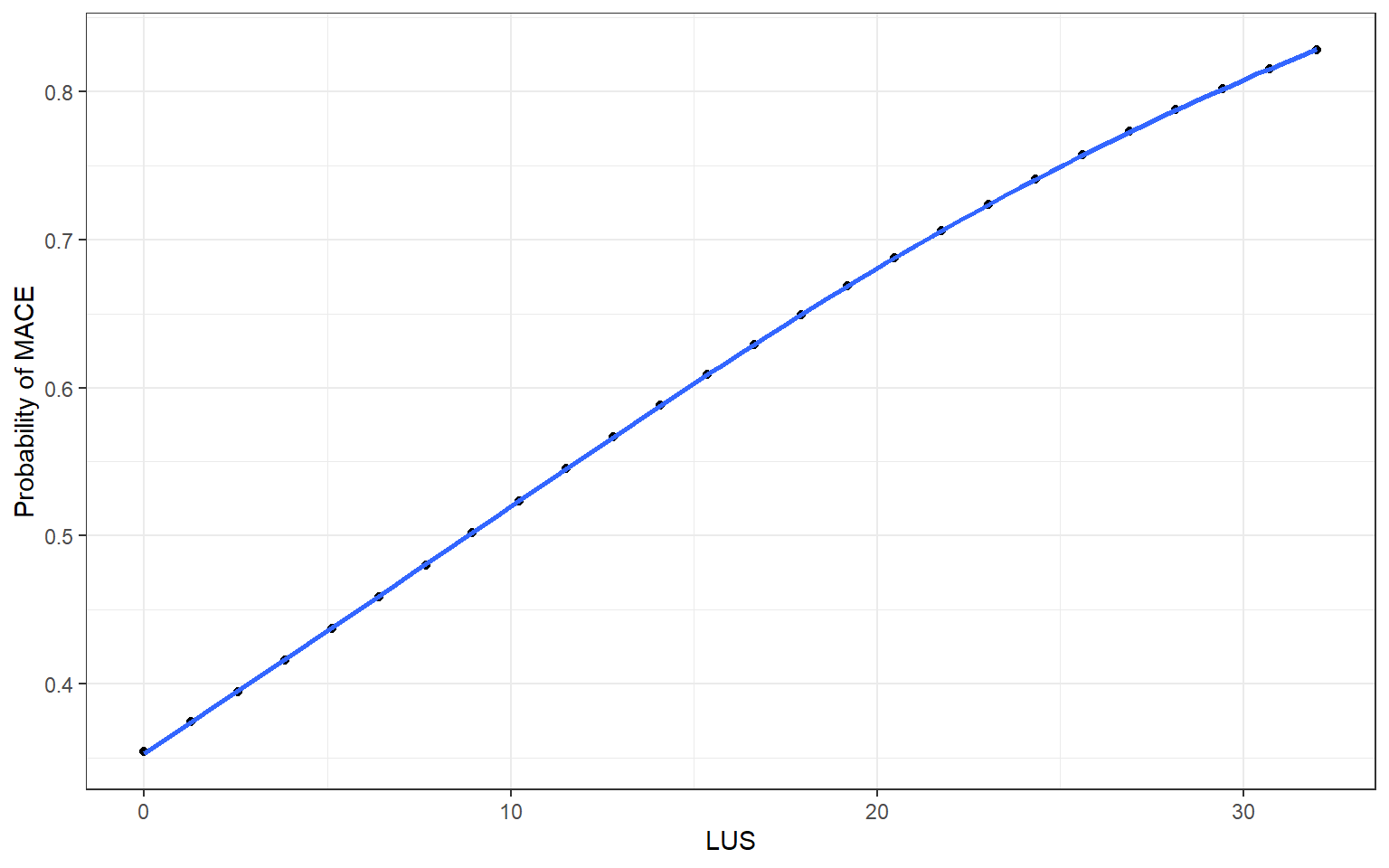* | *Panel B Partial Dependency Plot RCRI*  *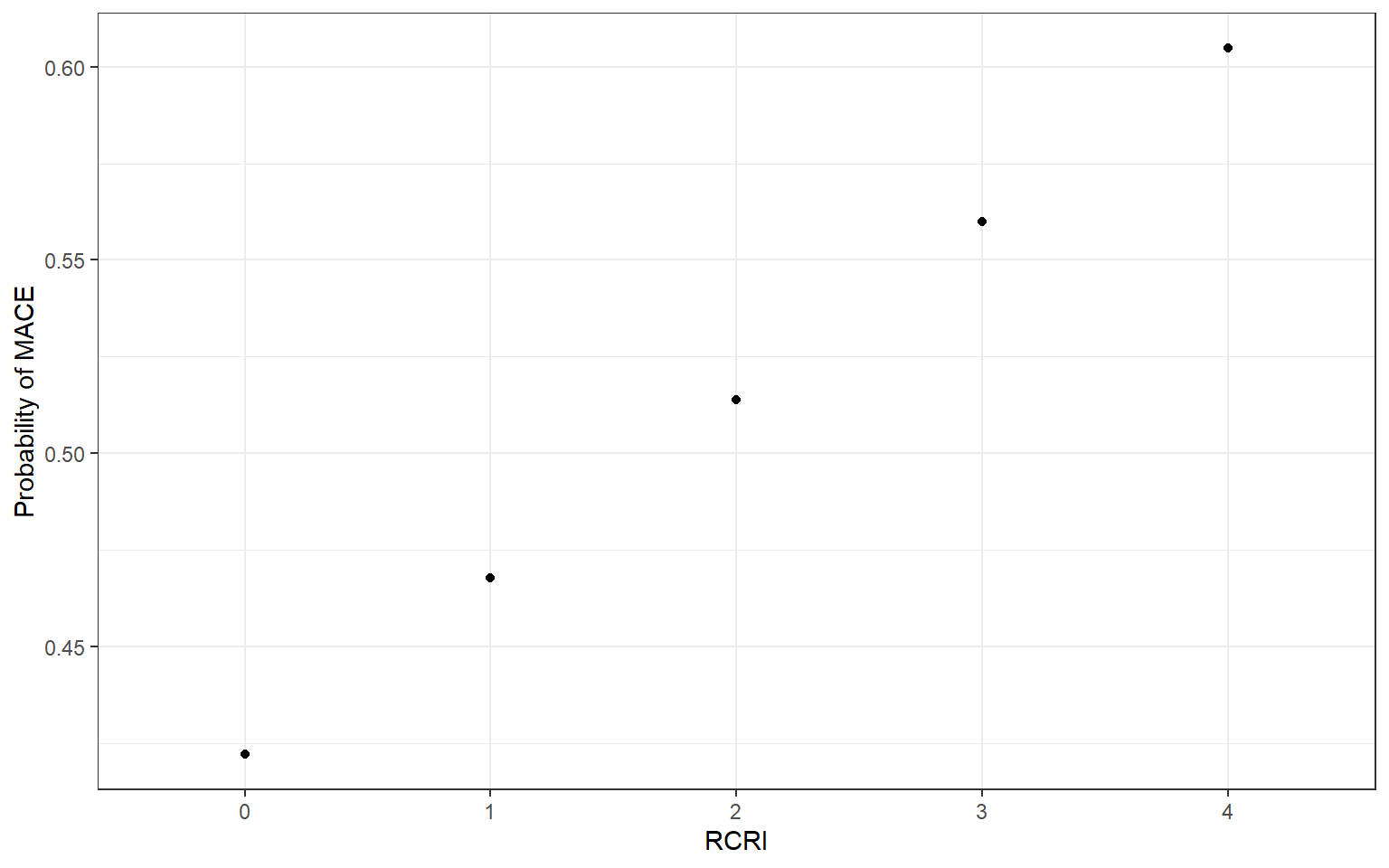* |
| --- | --- |

*Figure S6 Partial dependency plot for LUS (Panel A) and RCRI (Panel B) Score.*

## VIPOR Calculation

In our manuscript, we use an approach to assess variable importance using the VIPOR (Variable Importance for Projection Ordered Regression) method. This method focuses on evaluating the impact of different variables on the predictions made by our machine learning model, specifically tailored for variables of diverse types—binary, categorical, and continuous[10].

The core function, dynamically adjusts to the variable type under consideration, employing tailored strategies for binary variables, continuous variables with more than three levels, and categorical variables. For binary variables, the function manipulates the data frame to create two scenarios reflecting the binary outcomes and then computes the odds ratio (OR) based on the predicted probabilities from our model. This OR highlights the change in prediction odds when switching between binary outcomes, directly reflecting the variable's impact.

In handling continuous variables, the function identifies the 75th and 25th percentiles as representative values to gauge the variable's effect. By contrasting the model's predictions at these percentiles, we obtain an odds ratio that signifies the influence of varying levels of the continuous variable on the prediction odds.

For categorical variables, the approach is more nuanced, involving the comparison of each category against a reference, thereby generating a series of odds ratios that collectively map out the categorical variable's influence. This is achieved by systematically altering the data frame to reflect each category's presence and comparing it to a reference scenario, thereby allowing us to distill the effect of each category through the lens of prediction odds.


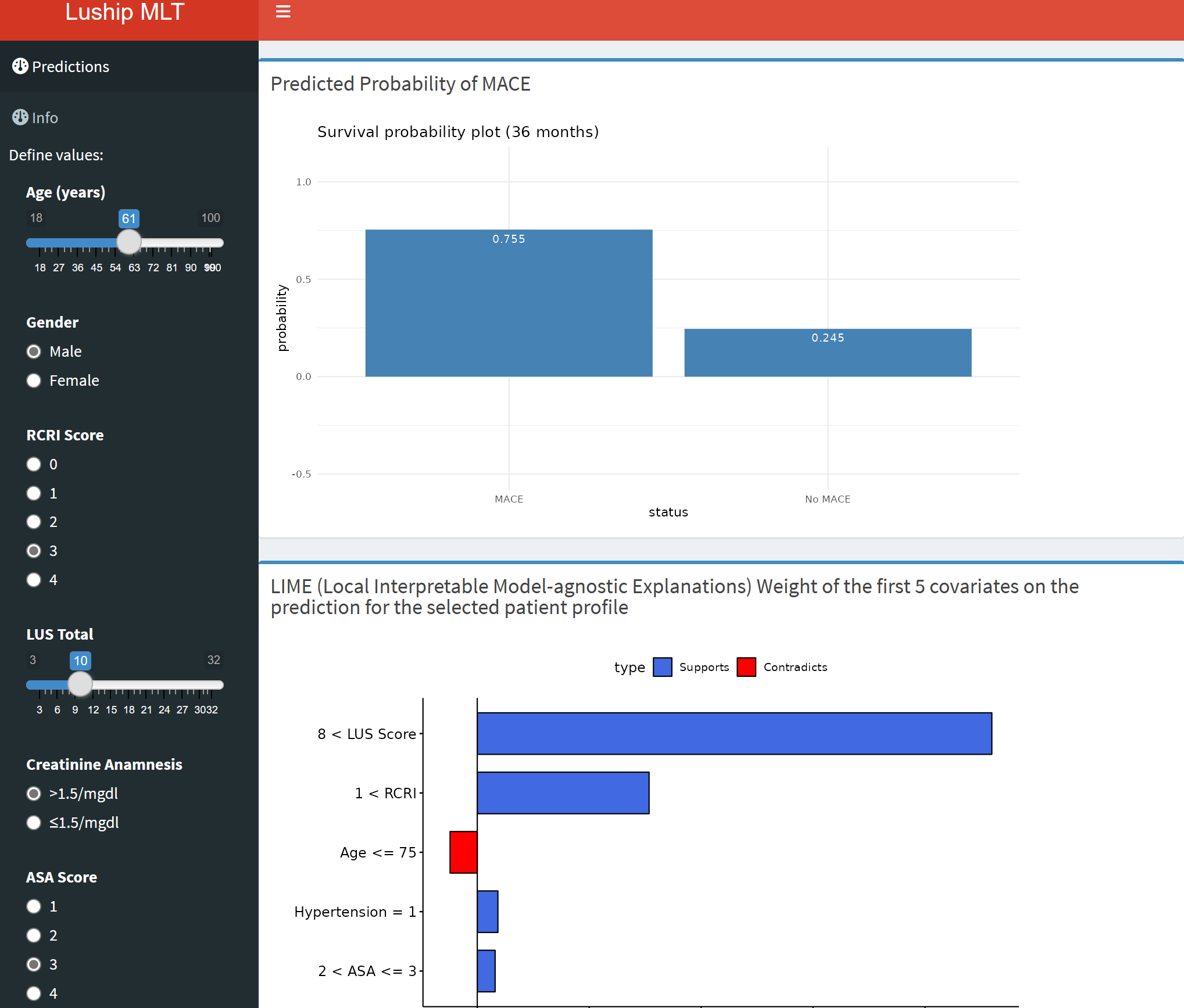


*Figure S7 Luship Web app*

## Bibliography

1. Breiman, L. Random Forest. *Machine Learning* **2001**, *45*, 5–32, doi:10.1023/A:1010933404324.

2. Friedman, J.H. Greedy Function Approximation: A Gradient Boosting Machine. *Ann. Statist.* **2001**, *29*, doi:10.1214/aos/1013203451.

3. Nelder, J.A.; Wedderburn, R.W.M. Generalized Linear Models. *Journal of the Royal Statistical Society. Series A (General)* **1972**, *135*, 370–384, doi:10.2307/2344614.

4. Zou, H.; Hastie, T. Regularization and Variable Selection via the Elastic Net. *J Royal Statistical Soc B* **2005**, *67*, 301–320, doi:10.1111/j.1467-9868.2005.00503.x.

5. Cortes, C.; Vapnik, V. Support-Vector Networks. *Machine Learning* **1995**, *20*, 273–297, doi:10.1007/BF00994018.

6. Scholkopf, B.; Burges, C.; Smola, A. Advances in Kernel Methods - Support Vector Learning. *MIT Press* **1998**.

7. Rumelhart, D.E.; Hinton, G.E.; Williams, R.J. Learning Representations by Back-Propagating Errors. *Nature* **1986**, *323*, 533–536, doi:10.1038/323533a0.

8. Haykin, S.S. *Neural Networks: A Comprehensive Foundation*; 2nd ed.; Prentice Hall: Upper Saddle River, N.J, 1999; ISBN 978-0-13-273350-2.

9. LeCun, Y.; Bengio, Y.; Hinton, G. Deep Learning. *Nature* **2015**, *521*, 436–444, doi:10.1038/nature14539.

10. Yu, D.; Wu, H. Variable Importance Evaluation with Personalized Odds Ratio for Machine Learning Model Interpretability with Applications to Electronic Health Records‐based Mortality Prediction. *Statistics in Medicine* **2023**, *42*, 761–780, doi:10.1002/sim.9642.
